# Synthesis, in vitro biological evaluation and molecular modelling of new 2-chloro-3-hydrazinopyrazine derivatives as potent acetylcholinesterase inhibitors‏ on PC12 cells

**DOI:** 10.1186/s13065-022-00799-w

**Published:** 2022-02-22

**Authors:** Maryam Taheri, Samira Aslani, Hossein Ghafouri, Asadollah Mohammadi, Vaha Akbary Moghaddam, Nastarn Moradi, Hananeh Naeimi

**Affiliations:** 1 Department of Biology, Faculty of Basic Sciences, University of Guilan, Rasht, Iran; 2 Department of Marine Sciences, The Caspian Sea Basin Research Center, University of Guilan, Rasht, Iran; 3 Department of Chemistry, Faculty of Sciences, University of Guilan, Rasht, Iran

**Keywords:** Acetylcholinesterase inhibitors, Alzheimer's disease, 2-Chloro-3-hydrazinopyrazine, PC12 cells

## Abstract

**Background:**

The loss of cholinergic neurotransmission in Alzheimer's disease (AD) patients' brain is accompanied by a reduced concentration of Acetylcholine (ACh) within synaptic clefts. Thus, the use of acetylcholinesterase inhibitors (AChEIs) to block the cholinergic degradation of ACh is a promising approach for AD treatment. In the present study, a series of 2-chloro-3-hydrazinopyrazine derivatives (CHP1-5) were designed, synthesized, and biologically evaluated as potential multifunctional anti-AD agents.

**Methods:**

In addition, the chemical structures and purity of the synthesized compounds were elucidated through using IR, ^1^H and ^13^C NMR, and elemental analyses. Further, the intended compounds were assessed in vitro for their AChE inhibitory and neuroprotective effects. Furthermore, DPPH, FRAP and ABTS assays were utilized to determine their antioxidant activity. The statistical analysis was performed using one-way ANOVA.

**Results:**

Based on the results, CHP4 and CHP5 exhibited strong AChE inhibitory effects with the IC_50_ values of 3.76 and 4.2 µM compared to the donepezil (0.53 µM), respectively. The study examined the effect and molecular mechanism of CHP4 on the Ab1–42-induced cytotoxicity in differentiated PC12 cells. At concentrations of 0–100 μM, CHP4 was non-toxic in PC12. Additionally, Ab1–42 significantly stimulated tau hyperphosphorylation and induced differentiated PC12 cell death. Further, CHP4 resulted in diminishing the Ab1–42-induced toxicity in PC12 cell significantly. CHP4 at 30 μM concentration significantly increased the Ab1–42-induced HSP70 expression and decreased tau hyperphosphorylation.

**Conclusions:**

According to the results of our studies CHP4 can be considered as safe and efficient AChEI and employed as a potential multifunctional anti-AD agent.

**Supplementary Information:**

The online version contains supplementary material available at 10.1186/s13065-022-00799-w.

## Background

AD is considered as one of the most causes of dementia and neurodegenerative disease among senile individuals, which may occur in 60% of cases. About 50 million individuals have dementia worldwide, to which approximately 10 million new cases are added every year. The total number of dementia individuals is expected to reach 82 and 152 million in 2030 and 2050, respectively [1, 2]. AD is characterized by the aggravation of cognition, function, and behavior, as well as losing memory. The growth of amyloid-β (Aβ) peptide deposits and hyperphosphorylated tau protein (Tau-p) in brain tissue has been addressed as the most important pathological hallmarks with AD [3]. In addition, heat shock proteins (HSPs) are an important class of molecular chaperones, which can involve under stress conditions such as hypoxia, oxidative stress, proteotoxic stress, inflammation, and several diseases like cancer, AD, and other neurodegenerative diseases. AD is associated with a type disorder of protein accumulation [4–6]. Further, HSP70 activation stabilizes Tau-p both in neuronal cells and brain tissues in the neurodegenerative diseases such as AD, Parkinson’s disease (PD), and epilepsy. HSP70 proteins are up-regulated in response to stress although it’s up-regulation can be damaging because of worsening the chronic inflammation [7–10].

Oxidative stress is detected as one of the causing factors in aging, which contributes to the development of multiple neurodegenerative disease such as AD, and plays an important role in destroying neuronal cells. Therefore, this process is considered as one of the primary incidents in AD. Accordingly, the effective protection of neuronal cells against oxidative stress damage can potentially prevent AD [11]. In AD, inflammatory processes generate reactive oxygen species (ROSs) leading to the dysfunction of antioxidant system [12]. AD is related to a decrease in ACh level in hippocampus (a part of medial temporal lobe memory system) and neocortex, especially in frontal and temporal lobes by cholinergic system deficiency such as the death or atrophy of basal forebrain cholinergic neurons [13]. Furthermore, the reduction of Ach synaptic availability is caused by basal forebrain cholinergic loss and memory deficits in AD [14]. Cholinesterases (ChEs) catalyze Ach hydrolysis into choline and acetate, which is an essential process in the cholinergic neurotransmission. Two types of ChEs are available, AChE (EC3.1.1.7) and Butyrylcholinesterase (BuChE, EC3.1.1.8), the first of which is one of the important factors in AD [15, 16]. AChE is involved in the termination of impulse transmission by hydrolyzing ACh in numerous cholinergic pathways in the nervous systems. Indeed, the active site of AChE is placed in the bottom of the molecule and contains anionic and esteratic subsites, and catalytic machinery and choline-binding pocket are related to esteratic and anionic subsites, respectively [17]. The catalytic machinery site is placed at the middle of the gorge and involves the catalytic triad (H447, E334, and S203) in human AChE. Further, the anionic site is formed by the side chains of Glu202, Trp86, and Tyr337, and considered as responsible for binding the ACh [18]. Some drugs such as tacrine, rivastigmine, donepezil, and galantamine have been consumed for years in most countries to diminish AD symptoms, which inhibit AChE. However, the number of AD patients is increasing and no definite treatment is currently available in this regard. There are many synthetic and natural inhibitors of AChE [19]. Furthermore, novel AChEIs have been designed and produced from a series of some 3,5-dimethoxy-*N*-methylenebenzenamine and 4-(methyleneamino) benzoic acid derivatives comprising of *N*-methylenebenzenamine nucleus (imin-metal) [20]. Pyrazine is one of the main types of heterocyclic compounds, synthesized chemically or extracted from natural sources [21]. So far, several synthesized derivatives of pyrazine have been introduced as important drugs and 2,5-dichloropyrazine is a basic starting compound for preparing many bioactive pyrazines [22, 23]. Pyrazine ring has become important in representing diverse biological activities in interaction with other scaffolds such as pyrrole, pyrazole, thiophene, oxazole, pyridine, triazole, tetrazole, imidazole, piperidine, and piperazine [24]. The presence of pyrazine ring as a vital scaffold in different clinically-used drugs exhibits its importance in drug design. Considering the above-mentioned reports, the present study sought to design and synthesize several novel 2-chloro-3-hydrazinopyrazine derivatives (CHP1-5) based on their pyrazine and formyl for assessing their inhibitory effects on the AChE in order to obtain new ligands with efficacy and high inhibitory effect. Then, the other biological evaluations such as antioxidant activity, enzyme, and cell protective assays were performed. The results suggested CHP4 as an effective AChEI. Among all compounds under study, CHP4 represented the highest antioxidant properties. Based on the results of anti-Alzheimer's studies and molecular docking ones on this compound, the protective effect of CHP4 against the Aβ-induced damage in PC12 cells was caused by changing Tau-p expression. In addition, the roles of HSP70 in the protective effect of CHP4 were considered in the present study. Finally, the results may propose the usage potential of CHP4 in subsequent research on AD.

## Methods

### Chemistry

Chemical materials, 2-chloro-3-hydrazinopyrazine; chromene; methoxy benzaldehyde; 3-hydroxybenzaldehyde; 2-hydroxybenzaldehyde; 4-chlorobenzaldehyde; glacial acetic acid and TLC-RP 18 (Silica gel 60) were purchased from Sigma-Aldrich.

#### Synthesis of the pyrazine-based Schiff-bases (CHP1-CHP5)

The 2-chloro-3-hydrazinopyrazine and formyl derivatives utilized to synthesize Schiff-bases and other chemicals were obtained from Sigma-Aldrich and Merck. Additionally, double-distilled water was consumed throughout the experiment and *n*-hexane–ethyl acetate solvent system was used as an eluent. Thin layer chromatography (TLC Silica gel 60 F254, Merck) was employed for determining the substrate purity and monitoring reaction. Further, an electrothermal melting point device was applied for specifying all melting points (mp). The visible spectra and FTIR ones in KBr pellets were respectively measured on a Pharmacia Biotech spectrophotometer and a Shimadzu 8400 FT-IR spectrophotometer. Furthermore, a FT-NMR (400 MHz) Brucker apparatus was used for recording ^1^H and ^13^C NMR spectra. Chemical shifts were expressed in δ ppm using TMS as an internal standard and coupling constants (*J*) were given in Hz.

The pyrazine-based Schiff-bases were prepared through the condensation reaction of 2-chloro-3-hydrazinopyrazine (0.5 mmol) with various formyl derivatives (0.5 mmol) in ethanol (15 mL) in high yield. As a typical procedure, 0.5 mmol 2-chloro-3-hydrazinopyrazine was added to a mixture of a formyl derivative (0.5 mmol), absolute ethanol (15 mL), and glacial acetic acid (organic catalyst) in a 50-mL round-bottom flask and heated to reflux with stirring for 7 h. The reaction progress was monitored through TLC using n-hexane: ethyl acetate (6:4) as solvent. After completing the reaction, the reaction mixture was cooled to room temperature, and the precipitate was collected by using vacuum filtration and washed with deionized water several times for affording the pyrazine-based Schiff-bases. Then, the products were isolated through recrystallization from DMF/H_2_O. Finally, the structure of the obtained Schiff-bases was confirmed by using FT-IR and ^1^H and ^13^C NMR spectroscopy.

#### (E)-2-chloro-3-(2-(4-methoxybenzylidene) hydrazinyl) pyrazine (CHP1)

Yellow solid, mp: 196–198 °C, yield: 87%, molecular weight (Mw): 262.69 g/mol, λ_max_ (DMF): 365 nm. FT-IR (KBr, cm^−1^): 610 (C–Cl), 1170 (C–N), 1242 (C–O), 1511 (C=C), 1647 (C=N), 3248 (N–H). ^1^H NMR (400 MHz, CDCl_3_, δ (ppm)): 3.86 (s, 3H, OCH_3_), 6.94 (d, 2H, CH, *J* = 8.8 Hz),7.73 (d, 2H, CH, *J* = 8.4 Hz),7.81 (dd,1H, CH *J* = 2.4 Hz), 8.07 (s,1H, CH), 8.21(dd,1H, CH, *J* = 2.4 Hz), 8.58 (s,1H, NH). ^13^C NMR (100 MHz, CDCl_3_, δ (ppm)): 55.39, 109.94, 114.20, 126.30, 129.04, 133.31, 141.52, 146.01, 147.52, 161.33.

#### (E)-3-((2-(3-chloropyrazin-2-yl)hydrazineylidene)methyl)-6-methyl-4H-chromen-4-one (CHP2)

Yellow solid, mp: 265–268 °C, yield: 85%, Mw: 314.76 g/mol, λ_max_ (DMF): 365 nm.

FT-IR (KBr, cm^−1^): 719 (C–Cl), 1170 (C–N), 1207 (C–O), 1518 (C=C), 1645 (C=N), 1665 (C=O), 3256 (N–H). ^1^H NMR (400 MHz, CDCl_3_, δ (ppm)): 2.51 (s, 3H, CH_3_), 7.46 (d,1H, CH, *J* = 8.8 Hz),7.56 (d,1H, CH *J* = 8.8 Hz), 7.88 (s,1H, CH), 8.09 (s,1H, CH), 8.23 (s,1H, CH), 8.41 (s,1H, CH), 8.74 (s, 1H, CH), 8.77 (s, 1H, NH). ^13^C NMR (100 MHz, CDCl_3_, δ (ppm)): 21.04, 118.30, 125.36, 125.4, 133.8, 134.1, 134.3, 135.50, 135.54, 136.05, 136.11, 138.51, 141.37, 153.98.

#### (E)-3-((2-(3-chloropyrazin-2-yl)hydrazineylidene)methyl)phenol (CHP3)

Yellow solid, mp: 210–215 °C, yield: 89%, Mw: 248.67 g/mol, λ_max_ (DMF): 365 nm.

FT-IR (KBr, cm^−1^): 689 (C–Cl), 1170 (C–N), (C–O), 1666 (C=N), 3291 (O–H), 3420 (N–H). ^1^H NMR (400 MHz, CDCl_3_, δ (ppm)): 6.80 (d,1H,CH, *J* = 8 Hz), 7.08 (d, 1H, CH, *J* = 7.6 Hz), 7.24 (m, 2H, CH), 7.85 (s,1H,CH), 8.23 (s, 1H, CH), 8.43 (s, 1H, CH), 9.59 (s, 1H, OH), 10.77 (s, 1H, NH). ^13^C NMR (100 MHz, CDCl_3_, δ (ppm)): 112.76, 117.35, 118.87, 130.32, 132.91, 133.79, 136.52, 141.97, 146.31, 148.37, 158.11.

#### (E)-2-((2-(3-chloropyrazin-2-yl)hydrazineylidene)methyl)phenol (CHP4)

Brown solid, mp: 140–144 °C, Mw: 248.67 g/mol, λ_max_ (DMF): 386 nm.

FT-IR (KBr, cm^−1^): 747 (C–Cl), 1053 (C–N), 1168 (C–O), 1666 (C=N), 3287 (O–H), 3420 (N–H). ^1^H NMR (400 MHz, CDCl_3_, δ (ppm)): 6.93 (d, 2H, CH, *J* = 9.2 Hz), 7.29 (t, 1H, CH, *J* = 7.6 Hz), 7.46 (d, 1H, CH, J = 7.6 Hz), 7.89 (s, 1H, CH), 8.27 (dd, 1H, CH, *J* = 1.6 Hz), 8.70 (s, 1H, CH), 11.2 (s, 1H, OH), 11.55 (s, 1H, NH). ^13^C NMR (100 MHz, CDCl_3_, δ (ppm)): 116.89, 119.32, 119.74, 130.09, 131.20, 132.98, 134.20, 142.08, 147.36, 147.86, 157.69.

#### (E)-2-chloro-3-(2-(4-chlorobenzylidene)hydrazineyl)pyrazine (CHP5)

Brown solid, mp: 201–204 °C, yield: 92%, Mw: 267.11 g/mol, λ_max_ (DMF): 390 nm.

FT-IR (KBr, cm^−1^): 707 (C–Cl), 1168 (C–O), 1487 (C=C), 1647 (C=N), 3207 (N–H). ^1^H NMR (400 MHz, CDCl_3_, δ (ppm)): 7.52 (d, 2H, CH, *J* = 7.2 Hz), 7.73 (d, 2H, CH, *J* = 7.2 Hz), 7.87 (dd, 1H, CH, J = 2 Hz), 8.26 (s, 1H, CH), 8.50 (s, 1H, CH), 10.92 (S, 1H, NH). ^13^C NMR (100 MHz, CDCl_3_, δ (ppm)): 128.73, 129.37, 133.01, 134.10, 134.23, 134.29, 142.01, 144.70, 148.29.

### In vitro assessments

#### AChE inhibition assay

The AChE inhibitory activity of all synthesized novel derivatives and their controls was evaluated through using the Ellman method. In addition, the AChE (Sigma, EC number 3.1.1.7, CAS number 9000.81.1) from Electrophorus electricus was utilized in the study [25]. Further, 4 µL of enzyme solution (1 ng commercial enzyme, Tris–HCl, pH 7.4, final concentration of 0.1 mg/mL), 60 µL of 15 mM ATCI in 40 µL of water, 500 µL of 5 mM DTNB in Buffer B (0.1 mg/mL Tris–HCl, pH 7.4, containing 100 mM K3PO4 and 1 mM EDTA), and 10 µL of compounds (0.1–10 µM) were poured in a 96-well plate. Each concentration was assayed in triplicate. Furthermore, all of the synthesized compounds (CHP1-5) and their bases were dissolved in DMSO. Finally, absorbance was recorded by using a Pharmacia Biotech Ultrospec 3000 UV–Vis spectrophotometer at 410 nm.

#### Antioxidant activity

The radical scavenging activity of prepared compounds was assessed through using ABTS decolorization assay and method reported by Re et al. [26] with few modifications. In this regard, ABTS solution was diluted with 80% ethanol to the absorbance of 0.70 ± 0.05 at 734 nm. After adding 100 µL of the obtained compounds in ethanol (1–30 µM) or ascorbic acid, absorbance was measured at exactly 20 min in the same wavelength. DPPH assay was based on the method provided by Ghafouri et al. [27] with some modifications. Additionally, the quenching of free radicals by novel synthesized compounds was evaluated spectrophotometrically (UV–Vis Pharmacia Biotech Ultrospec 3000) at 517 nm against the absorbance of DPPH radical. Further, ferric reducing/antioxidant power (FRAP) assay was performed according to the previous study with some modifications [27], the principle of which was based on reducing a ferric 2,4,6-tris(2-pyridyl)-1,3,5-triazine (Fe^3+^-TPTZ) to ferrous in the presence of prepared compounds. Finally, a standard curve was drawn by considering the different concentrations (10–1000 µM) of ferrous sulfate.

#### Cell viability

*Rattus norvegicus* pheochromocytoma PC12 cells (Pastor Institute, Iran) were cultured in the Dulbecco's Modified Eagle's Medium (DMEM) containing 10% fetal bovine serum (FBS) and 1% penicillin/streptomycin (Gibco) at 37 °C with 5% CO_2_. In order to examine the role of CHP4, PC12 cells were treated with the different concentrations (10–100 µM) of CHP4 for 24 and 48 h. In addition, Ab1-42 peptides were dissolved in distilled water and primarily incubated at 37 °C for 7 d before using for constructing pre-aggregation. Further, PC12 cells were incubated with 100 µM aggregated Ab1-42 for 24 h to make a cellular AD model in vitro. Furthermore, 50 µL of DMSO and 10 µL of 0.5 mg/mL MTT stock solution were added to each well containing about 1 mL of medium and the mixture was incubated for 4 h. Then, the plates were agitated on a plate shaker for 30 min and optical density was read at 570 nm by using an ELISA reader (Biotech). A slightly-modified MTT method of Jamalzadeh et al. [28] was employed in the experiments and the cell viability of the control groups not exposed to CHP4 or Ab1-42 was defined as 100%.

#### Western blot analysis

To this end, the cells were seeded in the 6-well plate and treated with 20 and 30 µM CHP4 for 24 h. Additionally, they were washed with cold PBS, followed by lysing with lysis buffer (20 mM Tris (pH 7.5), 150 mM NaCl, 1 mM EDTA, 1% NP-40, 0.5% sodium deoxycholate, 0.1% SDS, 50 mM Tris, protease and phosphatase inhibitor cocktails) for 30 min on ice. After centrifuging lysates at 4 °C in 12,000*g* for 10 min, the supernatants were transferred to new tubes. Protein concentration was detected through using the Bradford assay [29]. Further, the denatured proteins was resolved on SDS-PAGE, transferred onto PVDF membranes (Millipore, Billerica, MA, USA), and blocked with 5% nonfat dry milk in the PBS containing 0.1% Tween-20 (PBST). Then, the membranes were incubated with the rabbit primary antibodies against Tau-p, HSP70, and β-actin (as a control group) overnight at 4 °C, and exposed with the horseradish peroxidase (HRP)-conjugated second antibody for 2 h at room temperature. Finally, the density of relative protein bands was determined through densitometric scanning the blots by using Image-J program.

### In silico studies

#### Geometry optimization, MEP and molecular descriptors

Geometry optimization of all five compounds was performed at DFT level theory with B3LYP hybrid exchange–correlation energy functional method and 6-31G++ (d, p) basis by Gaussian 09 software [30]. From the optimized structures, the molecular electrostatic potential (MEP) of each compound was calculated by the same software and visualized using GaussView v. 6. The capability of molecules to permeate blood–brain barrier (BBB) was predicted by Online BBB Predictor [31] and other descriptors related to drug likeness property of each compound was calculated through using Padel descriptor [32].

#### Docking

AutoDock Vina software program was applied for docking CHP4 into hAChE protein [33]. In addition, the X-ray crystallographic structure of human AChE (PDB ID: 6O4W, 2.35 Å resolution) was obtained from the RCSB protein data bank (PDB). The protein was prepared through removing water, co-factor, and co-crystallized ligands by MolSoft ICM [34]. Further, the 2D structure of CHP4 was drawn using MarvinSketch (version 16.8.15) by ChemAxon and converted to 3D format by using molconverter from Jchem toolkit (http://www.chemaxon.com). All docking calculations were performed by considering the protein and ligand as inflexible and flexible, respectively. Furthermore, donepezil (DPZ) was docked into 6O4W as a positive control ligand to validate the docking protocol. Finally, the Vina output file was entered into ADT for analyzing the docking results, and the hydrophobic interactions of AChE-ligand complexes, as well as bond lengths were examined.

#### Analysis of binding sites and conserved sequences

For the purpose of identification of conserved sequences of AChE from an evolutionary aspect, ConSurf software was used with HMMER homolog search algorithm, MAFFT-L-INS-i method for alignments and Bayesian method for calculations [35].

### Statistical analysis

Each concentration was assayed in triplicates (n = 3) and repeated in three independent experiments. The values were expressed as mean ± SD and one-way ANOVA was utilized to determine the significant differences from controls statistically.

## Results and discussion

### Chemistry

#### Spectroscopic characterization of the pyrazine-based Schiff-bases

**Fig. 1:**
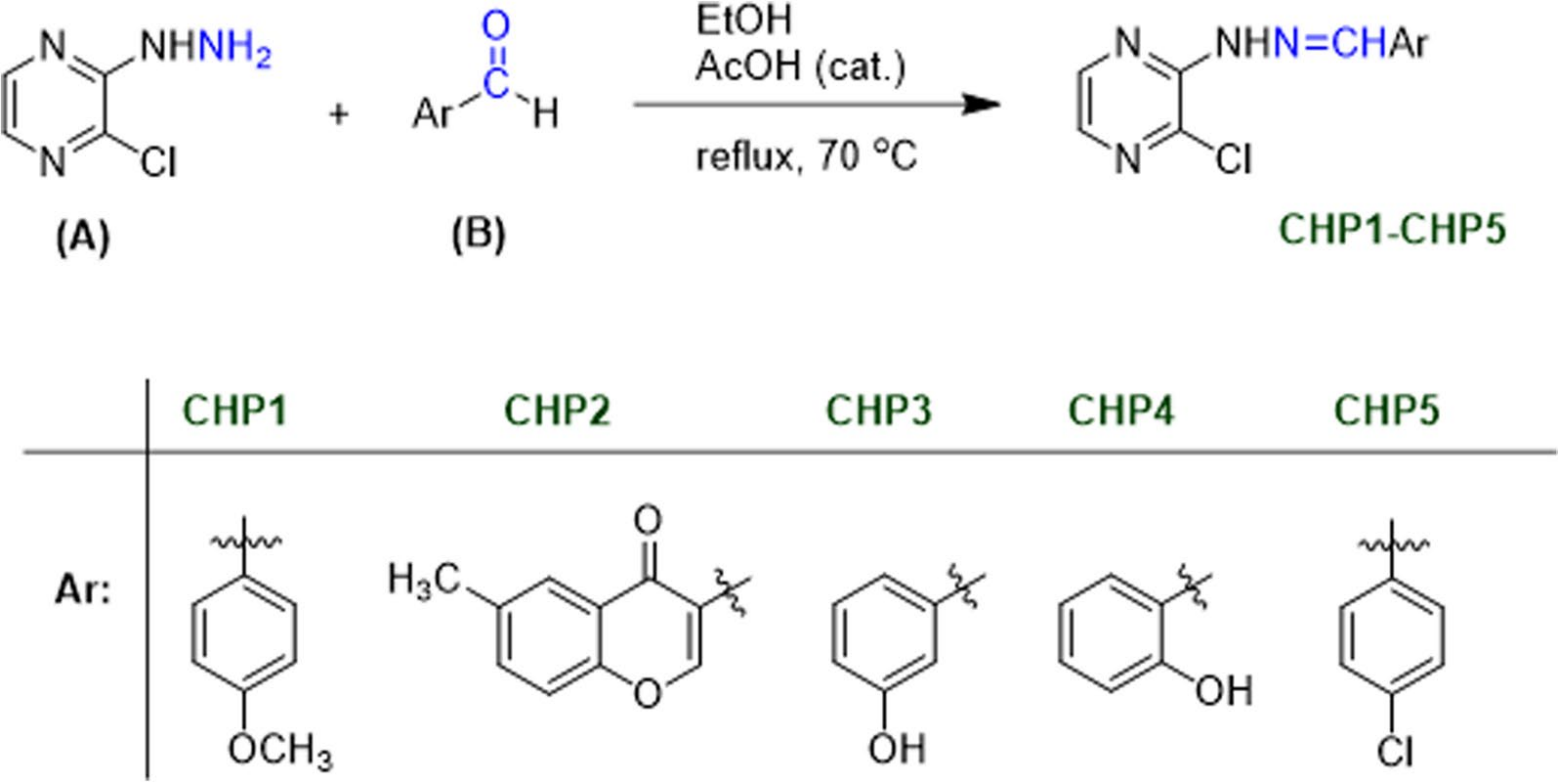
Synthesized pathways for the pyrazine-based compounds (CHP1-CHP5)

In the present study, five pyrazine-based Schiff-bases were synthesized through condensation reaction, the synthetic pathways for which are presented in Fig. 1. The chemical structure of compounds was approved by FT-IR, and ^13^C and ^1^H NMR spectroscopic data (see spectrums in Additional file 1). Additionally the UV–Vis spectra of all compounds were measured between 200 and 800 nm, which their λ_max_ was 365–390 nm due to n–π٭ and/or π–π٭ electronic transitions.

### In vitro

#### AChE inhibition assay

**Fig. 2:**
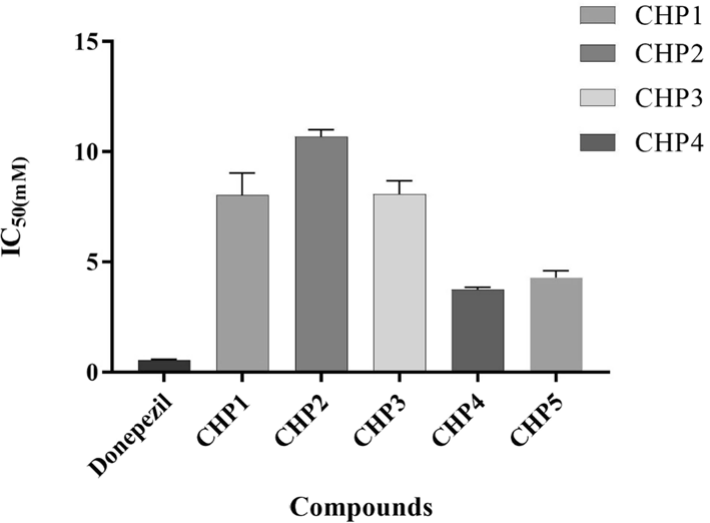
IC50 (µM) values of synthesized compounds against AChE activity (IC50 = 0.53 µM). CHP4 > CHP5 > CHP3 > CHP1 > CHP2. The data are expressed in relation to AChE activity with respect to control as mean ± SD (n = 3, P < 0.001). Donepezil is used as a positive control

All of obtained imine derivatives (CHP1-5) were evaluated for their inhibitory activities toward AChE through using an in vitro assay based on the reported protocol in comparison with donepezil as a standard drug (IC_50_ = 0.53 µM). The preliminary results in Fig. 2 demonstrated the highest AChE inhibitory effect in CHP4 with a hydroxyl group in the formyl (IC_50_ = 3.76 µM). In fact, the activity of the synthesized imine derivatives depends on the nature of the substituents attached to formyl group.

#### Antioxidant properties

**Fig. 3:**
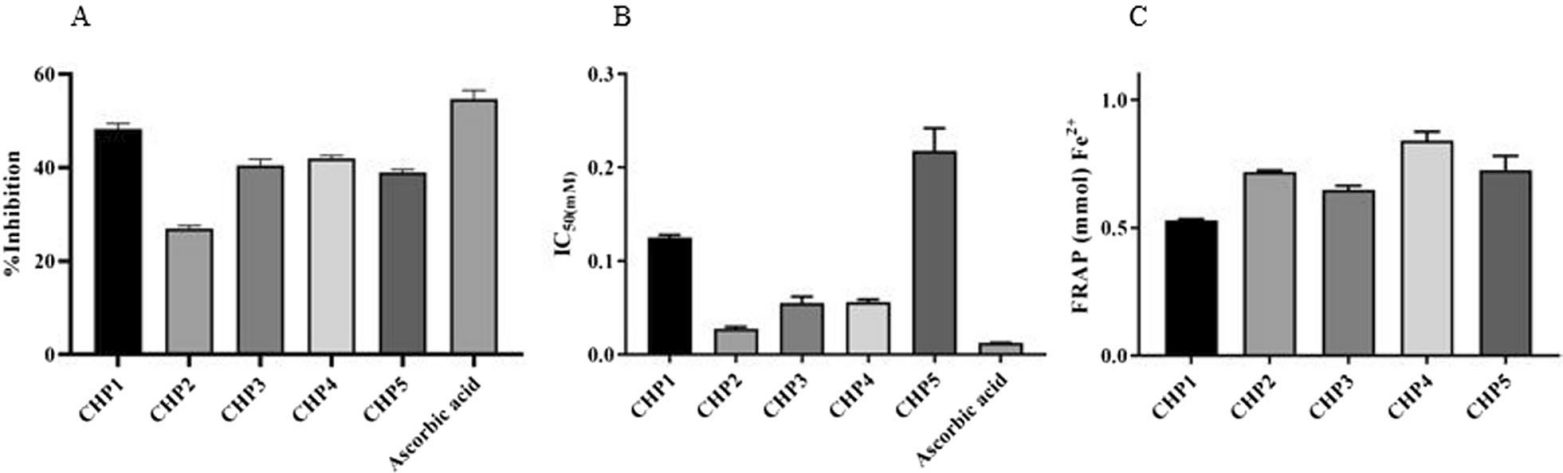
Antioxidant activities of synthesized compounds. Scavenging activity of prepared compounds on **A** ABTS radicals and **B** DPPH radicals (Ascorbic acid is used as a standard), and **C** concentration values of Fe^2+^ which indicate significant antioxidant properties in all of the synthesized compounds

The ABTS^+^ radical is created by oxidizing ABTS with potassium persulfate and reduced in the presence of such hydrogen-donating antioxidant compound. The results were obtained at the final concentration (1–30 μM) of compounds and their inhibition was assessed. The compounds were tested to determine by decolorizing the ABTS radical and assessed as a quenching of the absorbance at 734 nm. Based on the results, CHP1 exhibited high scavenging activity (48%) (Fig. 3A). Figure 3B compares the antiradical effect of compounds at various concentrations compared to that of ascorbic acid, which indicates the lower activity of all compounds. Further, the DPPH-reducing abilities of all compounds were measured by determining their IC_50_ values. As shown in Fig. 3B, the highest scavenging activity on DPPH^•^ are respectively observed in CHP2, CHP4, and CHP3, while CHP1 and CHP5 represent moderate and low properties, respectively. Furthermore, the antioxidant activity of all compounds was estimated by considering their ability to reduce TPRZ-Fe (III) complex to the TPTZ-Fe (II), the results of which are displayed in Fig. 3C. The concentration values of Fe^2+^ exhibited significant antioxidant properties in all synthesized compounds. Finally, the highest and least antioxidant effect was respectively obtained in CHP4 and CHP5.

#### Cell viability assays

**Fig. 4:**
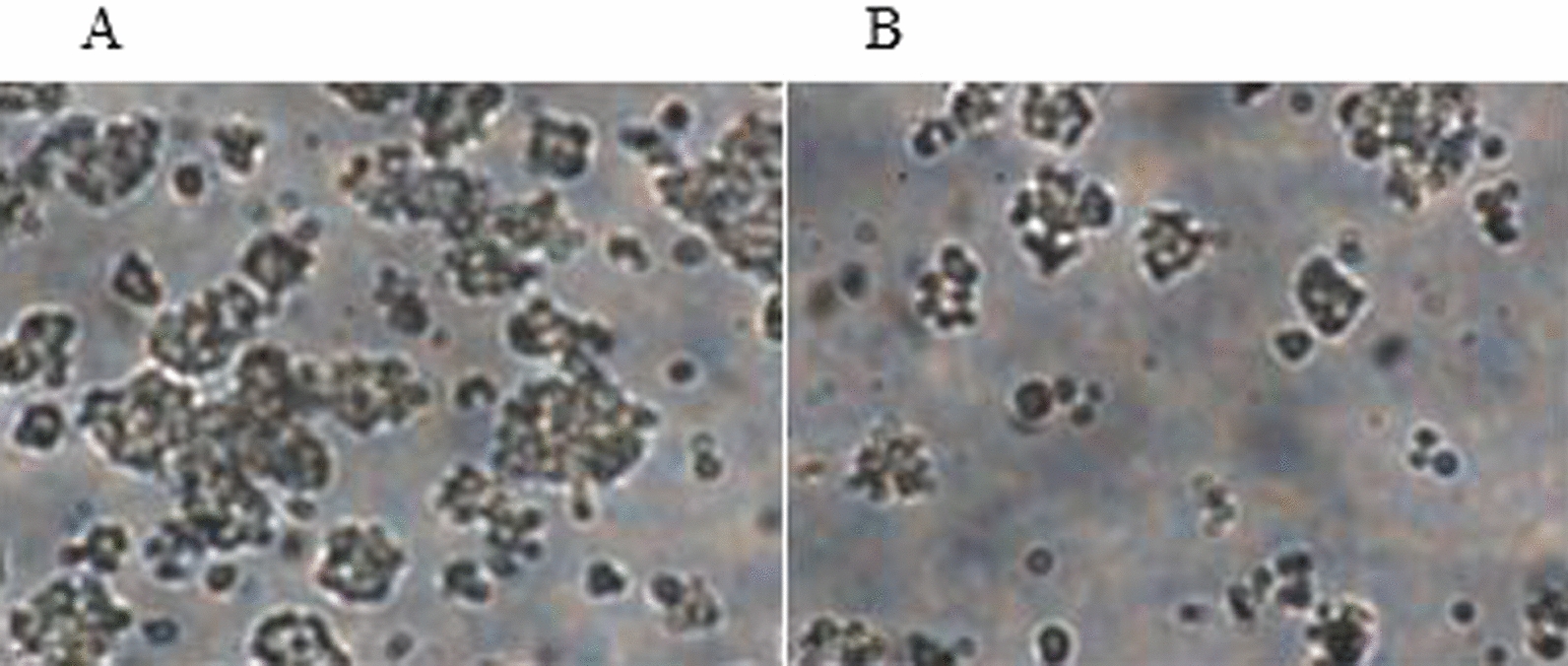
PC12 cells exposed to the Ab1-42 peptides in the **A** absence or **B** presence of 100 µM Ab1-42

**Fig. 5:**
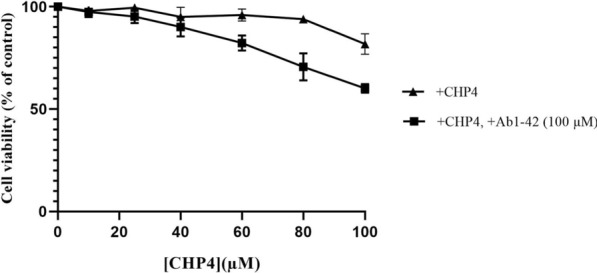
Protective effects of CHP4 at various concentrations against the Aβ1–42. Aβ1–42 (100 µM)-induced cytotoxicity in PC12 neuronal cells (Cell viability was assayed through using MTT. The values are presented as the mean ± SD of three independent experiments. The cell viability of the control group not exposed to either CHP4 or Ab1-42 is defined as 100%.)

In order to examine the neuroprotective effects of CHP4 against the Ab1-42-induced toxicity, PC12 cells were treated with CHP4 in the presence or absence of 100 µM Ab1-42 for 24 h. Then, cell viability was determined through using MTT assay. As depicted in Fig. 4, the viability of the PC12 cells exposed to 100 µM Ab1-42 for 24 h decreased to 49.24% compared to the control group. In addition, CHP4 significantly reduced the Ab1-42-induced cell death in a dose-dependent manner (Fig. 5). In the assays, the cell viability of the control group not exposed to CHP4 or Ab1-42 was considered as 100%.

**Fig. 6:**
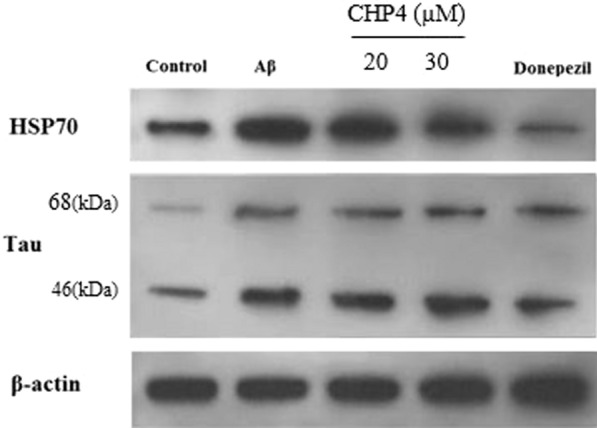
Representative western blot analysis of Tau-p and HSP70 in the PC12 cell treated with CHP4

In the pathological conditions, tau can be more phosphorylated than the normal phosphorylation, which is AD hallmarks [36]. Based on the western blot results of Tau-p and HSP70 in the PC12 cell treated with CHP4 and in the presence of Ab1-42, the level of tau phosphorylation increased significantly in the +Aβ1-42 group compared to the control. However, Tau-p expression clearly decreased in response to CHP4 at the final concentration of 20 and 30 µM. Also donepezil is utilized as a positive control (Fig. 6). Original photos of western blot gel are available, in Additional file 1.

### In silico studies

#### MEP

**Fig. 7:**
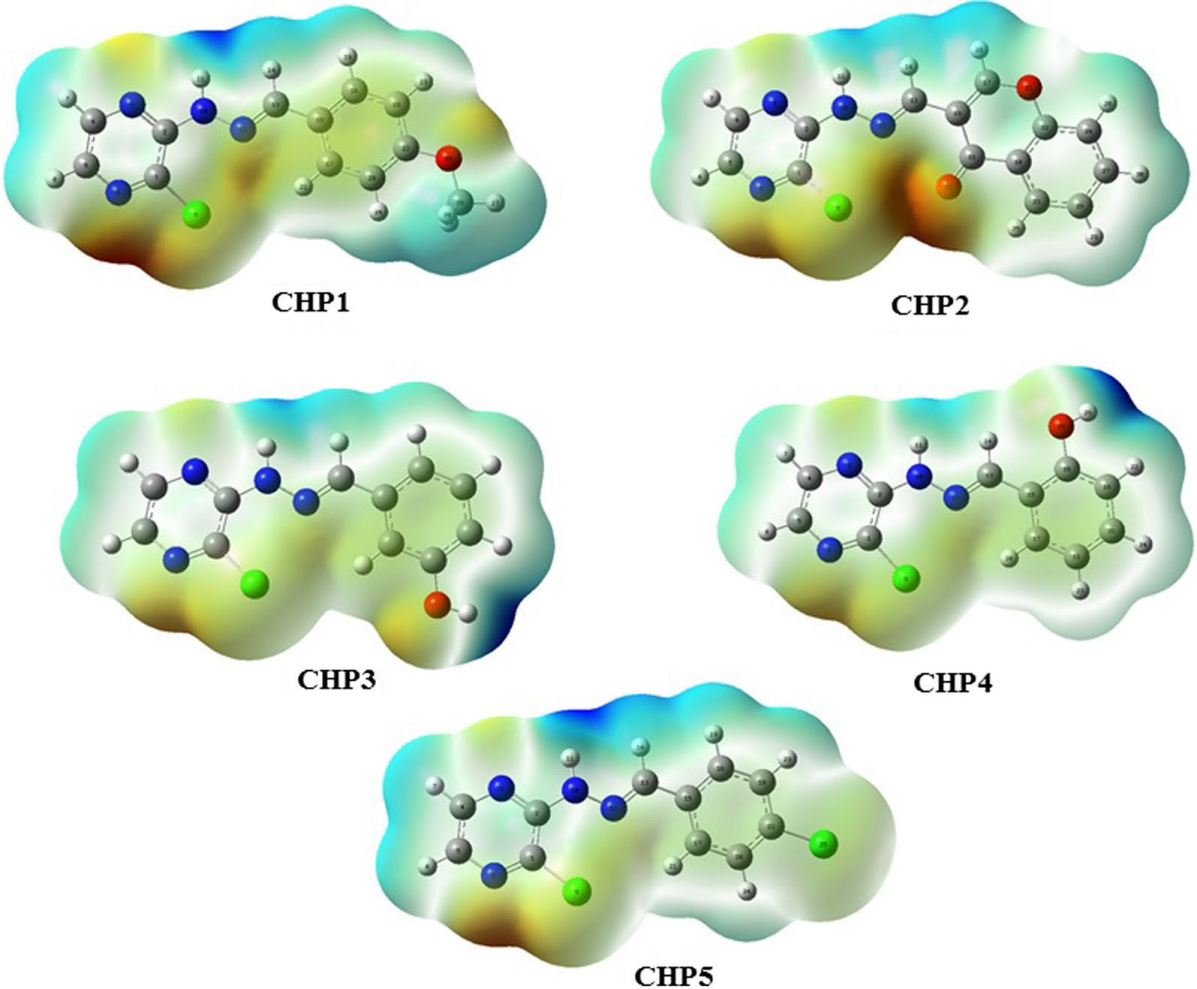
MEP of CHP1 to CHP5. For CHP1 MEP is represented from − 4.636 a.u. to + 4.636 a.u., for CHP2 it is from − 4.646 a.u. to + 4.646 a.u., for CHP3 from − 6.529 a.u. to + 6.529 a.u., for CHP4 from − 7.159 a.u. to + 7.159 a.u. and for CHP5 from − 5.104 a.u. to + 5.104 a.u. The most electron rich region for all compounds, except for CHP2, is located at N_6_ and for CHP2 it is O_19_. Furthermore, the least electron rich region for CHP1, CHP2 and CHP5 is at H_11_ but for CHP3 and CHP4 it is H_26_

First, the optimized structural properties of all compounds, which can be found in supplementary information, were obtained at DFT level of theory. The colored scheme of MEP visualization is presented in Fig. 7. The darkest red color indicates the most electron-rich region, capable of acting as one of the best hydrogen bond acceptors, whereas the darkest blue color represent the site which is the most sensitive towards nucleophilic attacks [37]. As can be seen in Fig. 7, except for CHP2 that has its most electron-rich region located at its carbonyl group, the same region is located at N_6_ of other compounds. Additionally, while CHP1, CHP2 and CHP4 have their least electron-rich regions at their N–H of hydrazine group, for CHP3 and CHP4 it is related to the phenolic hydrogen.

#### Drug likeness properties of the compounds

First, it was predicted that all compounds are capable of passing from BBB and their diagrams are presented in supplementary information. Lipinski’s rule of five was employed as the basis of investigating the drug likeness properties of compounds. According to this rule, an ideal drug candidate must not have more than 5 hydrogen bond donors, more than ten hydrogen bond acceptors, a molecular mass less than 500 Da and logP more than 5 [38]. As has been represented in Table 1, apart from the molecular mass, other criteria of the rule apply to the studied compounds.

**Table 1:** Drug-likeness properties of CHP1 to CHP4

Descriptor	CHP1	CHP2	CHP3	CHP4	CHP5
Hydrogen bond donor	1	1	2	2	1
Hydrogen bond acceptor	5	6	5	5	4
AlogP	0.027	0.054	− 0.380	− 0.380	0.82
Number of rotatable bonds	4	5	3	3	3
Polar surface area	59.4	76.47	70.4	70.4	50.17
Molecular mass	262.062	314.057	248.046	248.046	266.012
Number of atoms	29	33	26	26	25

#### Molecular docking of CHP4 and donepezil

**Fig. 8:**
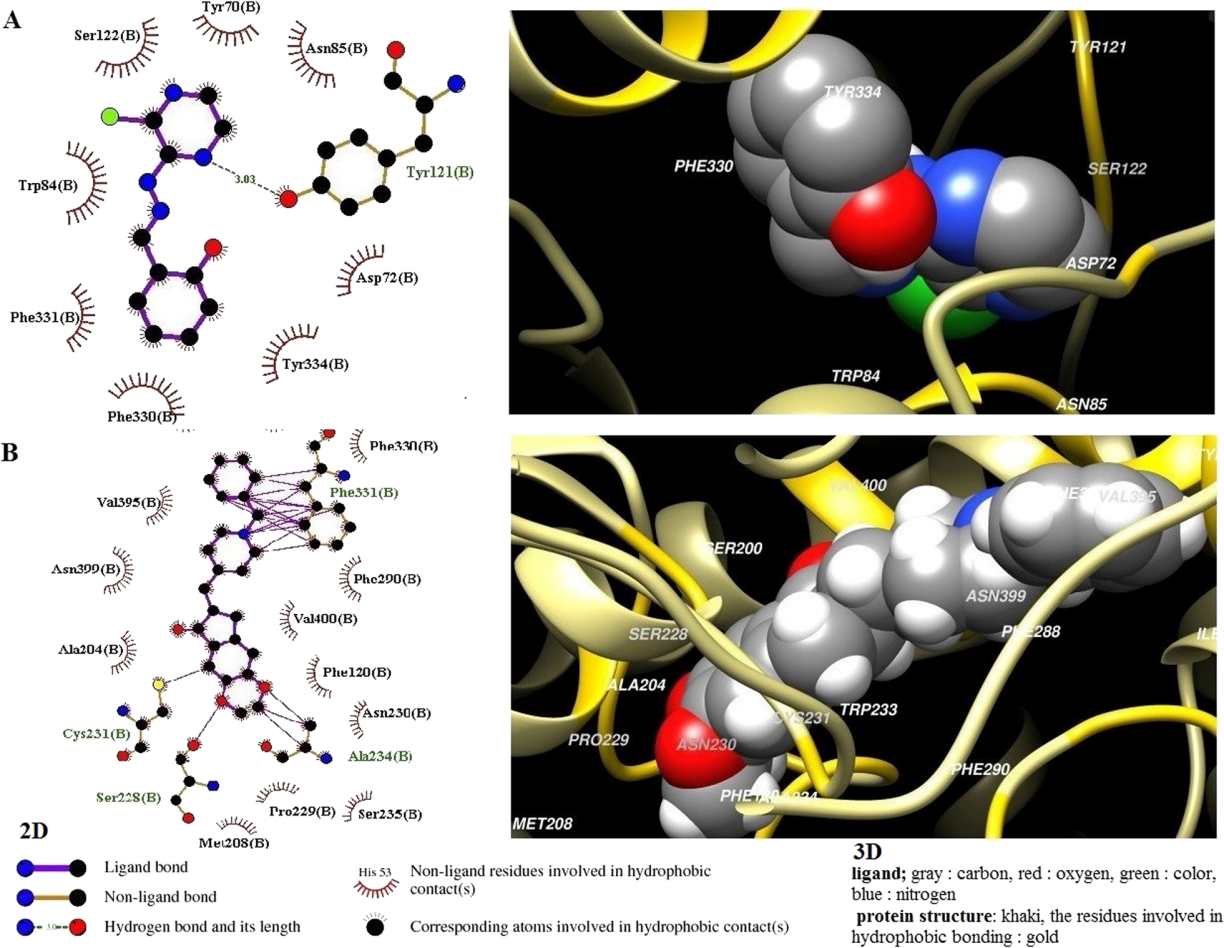
2D (left) and 3D (right) illustration of the complexes of 6O4W with CHP4 (**A**) and DPZ (**B**). In the 3D-interaction diagram, the ligand is represented as a solid and protein as a ribbon

Following the end of docking process, the best conformation was selected and the scoring functions indicated that conformations were the best complement to the AChE. Additionally, AutoDock 4.2 software was applied for all of the docking calculations (see vina results in the Additional file 1). The docking of CHP4 affinity with active site was calculated as − 8.3 kcal/mol. D72, W84, N85, Y121, S122, F330, Y334 and F331 were the residues interacting with CHP4. Moreover, the evolutionary analysis of AChE revealed that out of this residues, Y121 is highly conserved. In order to validate the docking results obtained by Vina software, donepezil was docked in 6O4W as positive control ligand (RMSD: 0.307 Å) with an affinity of − 12.8 kcal/mol (Table 2). After docking, the interaction of CHP4 with AChE was evaluated by using UCSF Chimera [39] and LigPlot [40] software (Fig. 8). More figures are available in the additional file. Finally.

**Table 2:** List of amino acids involved in hydrogen bonding and hydrophobic interactions with CHP4 and donepezil with AChE

Compounds	UCSF Chimera		LigPlot+	
	H-bonds	HI	H-bonds	HI
CHP4	S124*b*	*Chain b:* W84, Y121, D72, S122, *F331, *Y334, *F330, N85	Y121*b*	*Chain b:* W84, Y121, D72, S122, *F331, *Y334, *F330, N85
DPZ	S228*b,* S205*b*	*Chain b:* F288, A234, W233, *F331, C231, S288,A204, P229, F290, N230, F120, M208, S200, I287, *F330, N399, S235, V395, L332, *Y334, V400	_	*Chain b:* F288, A234, *F331, C231, S288,A204, P229, F290, N230, F120, M208, *F330, N399, S235, V395, L332, *Y334, V400

HI: Hydrophobic Interactions *Common residue

## Discussion

Designing, synthesizing, and producing the molecules valuable as human therapeutic agents are considered one of the principal purpose of pharmaceutical and organic chemistry. Pyrazines possess various types of biological activity, which represents a range of their pharmacological activities such as antibacterial, antimycobacterial, antiviral, antifungal, anti-diabetic, anti-cancer, analgesic, hypnotic, diuretic [23], and anticholinergic ones [41, 42]. In the present study, several novel 2-chloro-3-hydrazinopyrazine derivatives were designed, synthesized, and evaluated, which their pyrazine base was effective (Fig. 1). Pyrazine is an aromatic heterocyclic ring, which contains two nitrogen atoms with the general effect of protonation and hydrogen-bond formation [43]. The molecular characteristics of all of the synthesized compounds (Table 1), indicated that these molecules have potential as drug candidates. In the AD therapy, one of the aims of treatment is to inhibit AChE [44]. Accordingly, some novel compounds have been produced for assessing the inhibitory effects on AChE. In this study, the inhibitory effects of the novel compounds on AChE were assessed and the relationship between AChE inhibitory activity with the chemical structures of the compounds was evaluated. Based on the preliminary results, CHP4 with a hydroxyl group at the ortho position of *N*-phenyl ring exhibited the highest AChE inhibitory effect (IC_50_ = 3.76 µM). The effectiveness of CHP4 may be related to the electron withdrawing property of the phenyl ring [45]. Computational analysis illustrated that CHP4 is capable of having hydrogen interaction with Tyr121 through its nitrogen of pyrazine ring (Fig. 8), which is the most electron-rich region of the compound according to MEP and the same residue in the protein is a highly conserved sequence from an evolutionary aspect. According to Uysal et al., the formation of mono- and di-substituents to the ortho position of *N*-phenyl ring improved AChE inhibitory effect slightly [46]. Furthermore the hydroxyl group was not involved in hydrogen bonding with AChE, in spite the fact that its hydrogen possesses the least electron-rich region of the molecule based on MEP [47].

In the AD patients, the use of antioxidant therapies for the disease is necessary to reduce pathogenic symptoms [48]. The results of the present study demonstrated that CHP2 represented the most potent antioxidant properties (IC_50_ = 0.026 mM) in the DPPH assay due to its higher capacity to donate electron compared to the others (CHP2 > CHP3 > CHP4 > CHP1 > CHP5). In the ABTS assay, CHP1 performed better than the others due to the presence of electron-withdrawing and electron-donating group, methoxy group, with 48% inhibition (CHP1 > CHP4 > CHP3 > CHP5 > CHP2). Furthermore, FRAP assay is based on the ability of antioxidants to reduce Fe^3+^ to Fe^2+^. The highest ability for Fe^3+^ reduction was observed in CHP4 (3.016 per 1 mM) (CHP4 > CHP2 > CHP5 > CHP3 > CHP1). The ortho position of a hydroxyl group on benzoic ring exhibited excellent antioxidant activity, while the meta position led to weak effect [49]. Based on previous studies and due to the toxicity of iron and its important role in the progression of AD [50–52], CHP4 was selected for following the study.

AD is a common neurodegenerative diseases, the hallmark pathologic characteristics of Aβ plaques, tau hyperphosphorylation, and neuronal cell death [2]. HSP70 and some of the other co-chaperones are involved in regulating, phosphorylation, aggregation and degradation of tau, and potentially implicated in pathogenesis of AD [53–56]. In the present study, the protective effect of CHP4 was examined against Ab1-42 toxicity in PC12 neuronal cells, the results of which reflected that CHP4 at therapeutic-relevant concentration protected and rescued neuronal cells from the toxicity of Ab1-42 peptide.

In addition, the effects of CHP4 on the expression of Tau-p and HSP70 were assessed using western blot analysis, which demonstrates that the exposure of CHP4 resulted in decreasing the Ab1-42-induced phosphorylation of tau and increased HSP70 expression. According to Lu et al., HSP700 plays cytoprotective roles in AD, blocks Aβ self-assembly, moderates caspase-dependent and caspase-independent apoptotic pathways and reduces it in neuron cells, it also directly prevents tau aggregation and enhance Aβ clearance by upregulates the expression of insulin degrading enzyme and transforming growth factor beta [57]. Some studies have reported the protective role of HSP70 in the CNS, while several others have found the pathological and detrimental role of HSP70 in AD. In fact, HSP70 acts as a double-sided sword and its role in AD is still unclear and controversial. According to Miyata et al., HSP70 is considered as an emerging pharmaceutical target for treating neurodegenerative tauopathies [58].

## Conclusion

In summary, five compounds (CHP1–CHP5) were designed, synthesized, and evaluated. In the following, all synthesized compounds were analyzed and identified by using IR, 1H and 13C NMR. Novel compounds have been assayed for assessing the inhibitory effects on AChE, which CHP4 was effective (IC50 = 3.76 µM). According to the results of DPPH, ABTS and FRAP assays, CHP4 was chosen for subsequent the study. We have shown that CHP4 has a protective effect on PC12 neuronal cells against the toxicity of Ab1-42 peptide. In supplemental studies of western blot analysis, CHP4 reduce the expression of Tau-p as an important cause to AD On the other hand, a decrease in HSP70 expression was also seen, which could be a sign of a diminish in the pathological symptoms of this disease.

The multifunctional properties (an optimal strategy) highlight CHP4 as a promising candidate for further studies on the development of novel drugs against AD. However, more extensive research should be conducted to assess the exact mechanism of effectiveness, as well as dose and potential in medical applications.

## Supplementary Information

**Additional file 1:** **Figures S1-S20**. 2D Structure image and 1H-NMR, 13C-NMR, FT-IR spectra of compounds CHP1, CHP2, CHP3, CHP4 and CHP5. **Figures S21-S23**. Original photos of western bot of CHP4. **Tables S1-S5**. Optimized geometric properties of CHP1 to CHP5. **Tables S6-S7**. Vina docking results. **Figures S24-S28**. Diagram of the capability of the synthesized compounds in passing from BBB. **Figures S29-S30**. Protein and ligand interaction, ribbon model of the dimeric structure (S29), and binding pocket (S30) of AChE.

## Data Availability

The datasets generated and/or analysed during the current study available from the corresponding author on reasonable request. We have presented all our principal data in the form of tables, figures and supplementary.
