# Supplementary material for: Synthesis, in vitro biological evaluation and molecular modelling of new 2-chloro-3-hydrazinopyrazine derivatives as potent acetylcholinesterase inhibitors‏ on PC12 cells
Source: BMC Chem. 2022 Feb 22;16(1):7. doi: 10.1186/s13065-022-00799-w (PMC8864858; doi:10.1186/s13065-022-00799-w)
Supplement: Supplementary file 1 — Additional file 1: Figures S1-S20. 2D Structure image and 1H-NMR, 13C-NMR, FT-IR spectra of compounds CHP1, CHP2, CHP3, CHP4 and CHP5. Figures S21-S23. Original photos of western bot of CHP4. Tables S1-S5. Optimized geometric properties of CHP1 to CHP5. Tables S6-S7. Vina docking results. Figures S24-S28. Diagram of the capability of the synthesized compounds in passing from BBB. Figures S29-S30. Protein and ligand interaction, ribbon model of the dimeric structure (S29), and binding pocket (S30) of AChE. [file 13065_2022_799_MOESM1_ESM.docx]

**Synthesis, in vitro biological evaluation and molecular modelling of new 2-chloro-3-hydrazinopyrazine derivatives as potent acetylcholinesterase inhibitors‏ on PC12 cells**

*Maryam Taheri^1^, Samira Aslani^1^, Hossein Ghafouri***^1^, Asadollah Mohammadi^2^, Vaha Akbary Moghaddam ^1^ and Hananeh Naeimi^1^*

*^1^ Department of Biology, Faculty of Basic Sciences, University of Guilan, Rasht, Iran*

*^2^Department of Chemistry, Faculty of Sciences, University of Guilan, Rasht, Iran*

* Corresponding author: E-mail: h.ghafoori@guilan.ac.ir Tel/Fax: +9813333333647

**Structure image and ^1^H-NMR, ^13^C-NMR, FT-IR spectra of compounds CHP1, CHP2, CHP3, CHP4 and CHP5.**

***(E)-2-chloro-3-(2-(4-methoxybenzylidene)hydrazinyl)pyrazine(CHP1)***


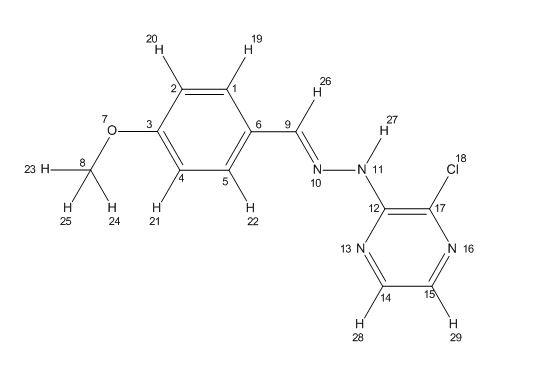


**Fig. S1** Structure image of compounds CHP1.


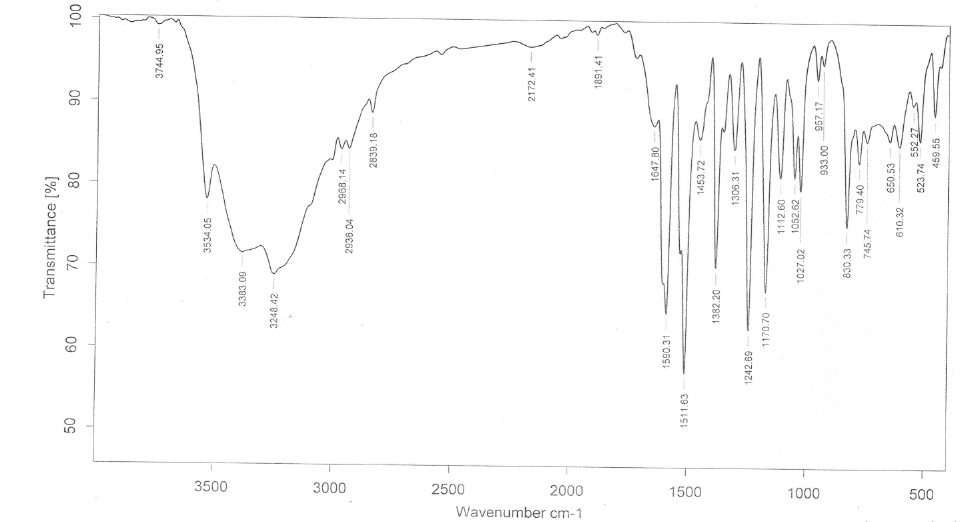
**Fig. S2** FT-IR spectra of compounds CHP1.

**Fig. S3** ^1^H-NMR, spectra of compounds CHP1.

**Fig. S4** ^13^C-NMR spectra of compounds CHP1.

***(E)-3-((2-(3-chloropyrazin-2-yl)hydrazineylidene)methyl)-6-methyl-4H-chromen-4-one (CHP2)***


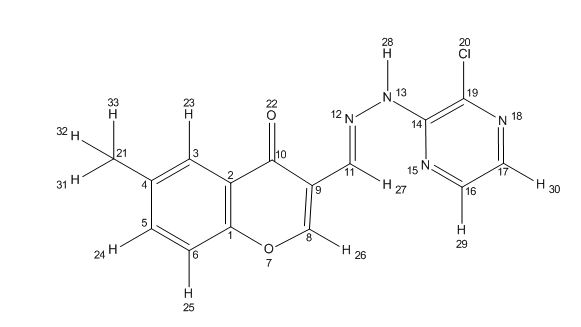


**Fig. S5** Structure image of compounds CHP2.

 **Fig. S6** FT-IR spectra of compounds CHP2.

 **Fig. S7** ^1^H-NMR, spectra of compounds CHP2.

**Fig. S8** ^13^C-NMR spectra of compounds CHP2.

***(E)-3-((2-(3-chloropyrazin-2-yl)hydrazineylidene)methyl)phenol (CHP3)***


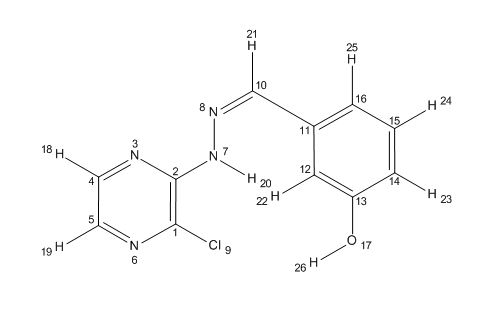


**Fig. S9** Structure image of compounds CHP3.

 **Fig. S10** FT-IR spectra of compounds CHP3.

**Fig. S11** ^1^H-NMR, spectra of compounds CHP3.

**Fig. S12** ^13^C-NMR spectra of compounds CHP3.

***(E)-2-((2-(3-chloropyrazin-2-yl)hydrazineylidene)methyl)phenol (CHP4)***


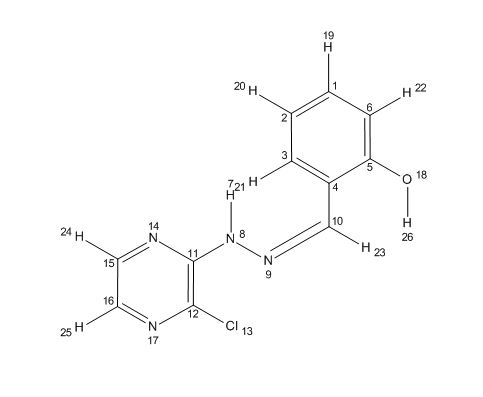


**Fig. S13** Structure image of compounds CHP4.

**Fig. S14** FT-IR spectra of compounds CHP4.

 **Fig. S15** ^1^H-NMR, spectra of compounds CHP4.


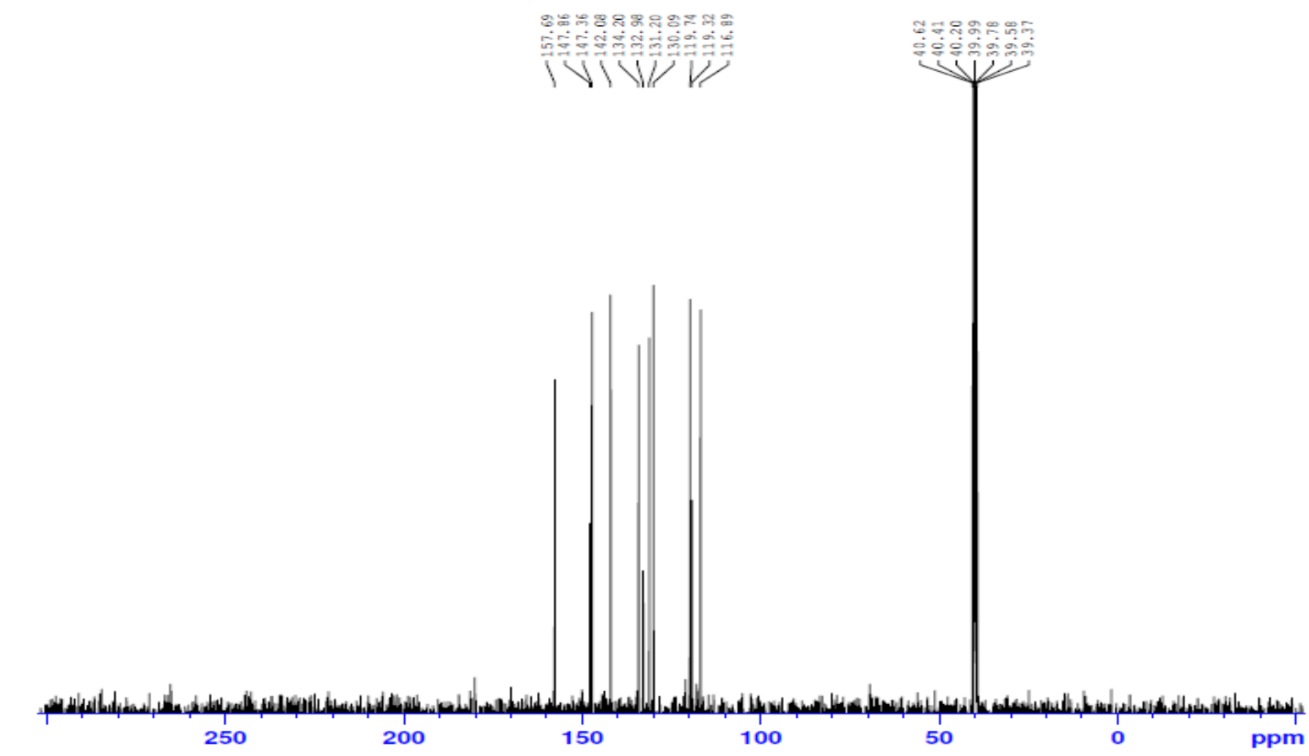


**Fig. S16** ^13^C-NMR spectra of compounds CHP4.

***(E)-2-chloro-3-(2-(4-chlorobenzylidene)hydrazineyl)pyrazine (CHP5)***


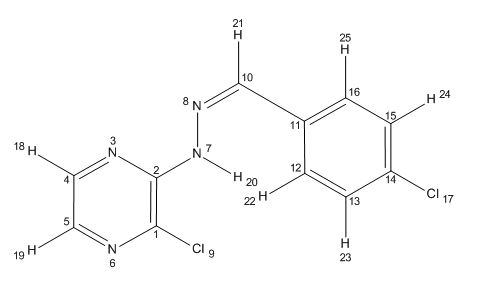


**Fig. S17** Structure image of compounds CHP5.


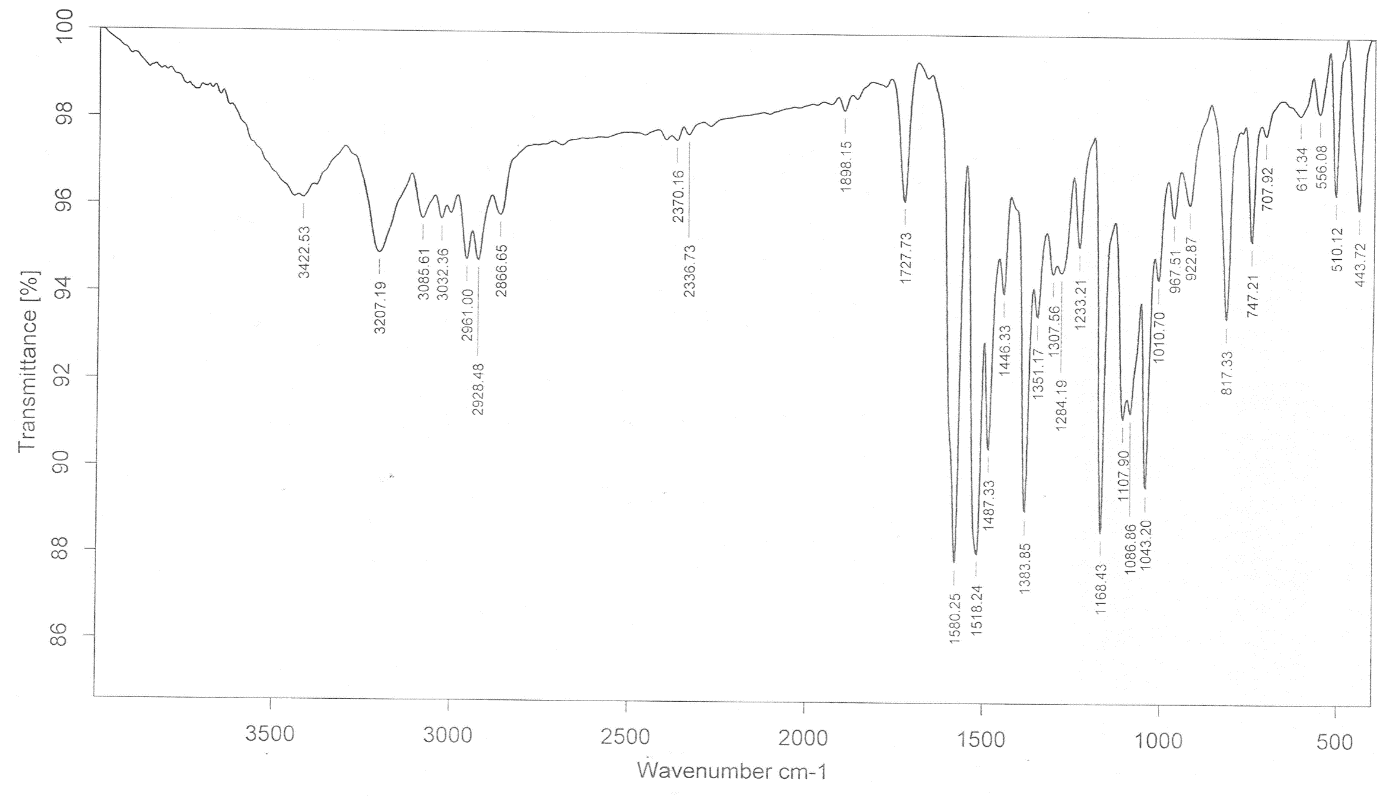


**Fig. S18** FT-IR spectra of compounds CHP5.


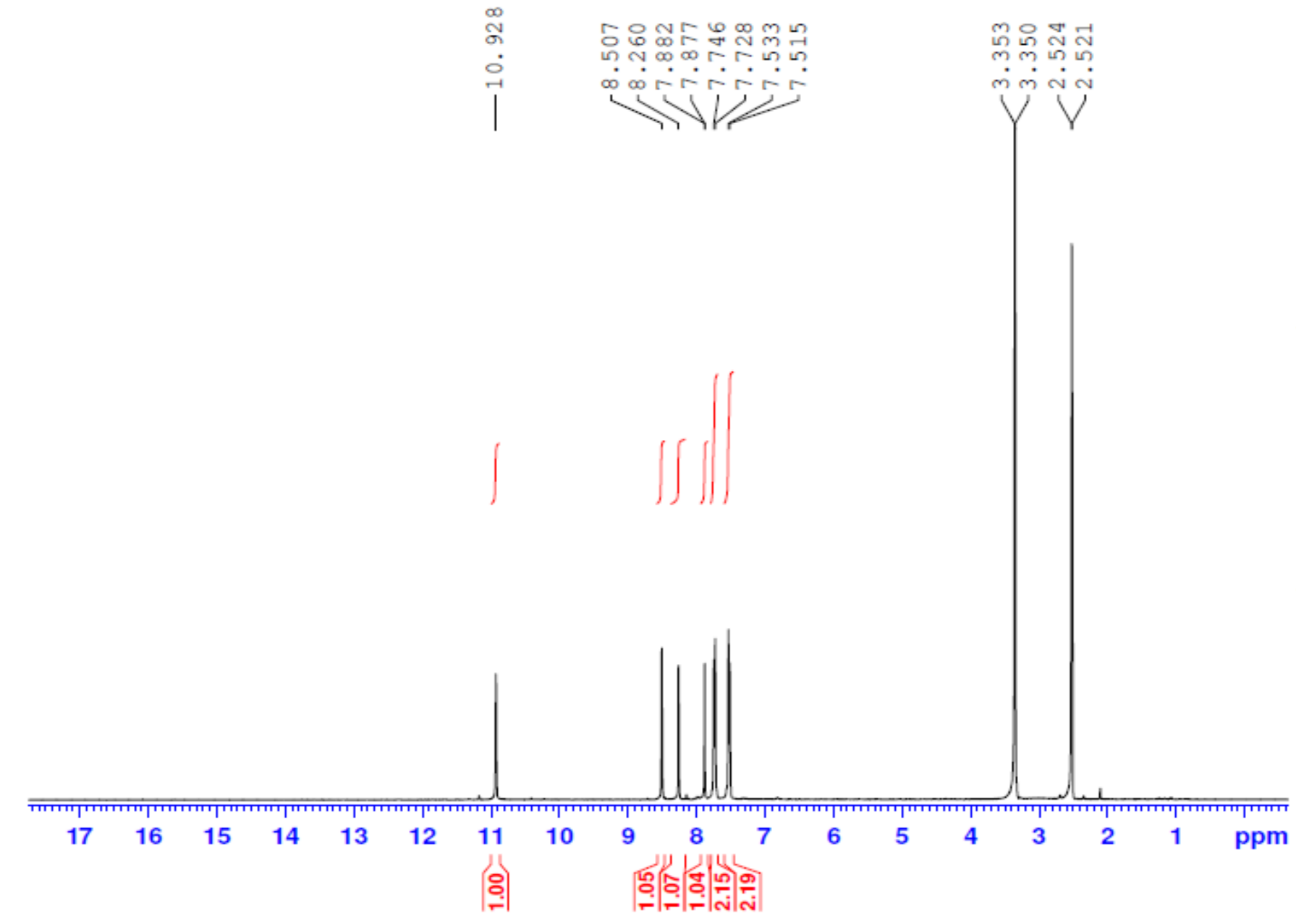


**Fig. S19** ^1^H-NMR, spectra of compounds CHP5.


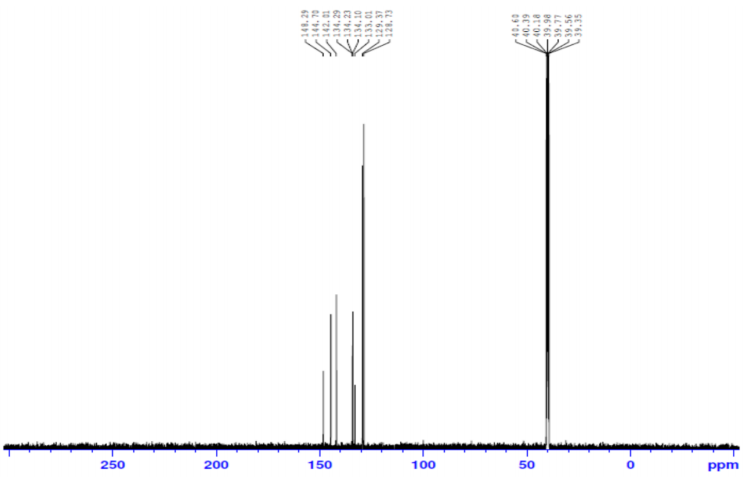


**Fig. S20** ^13^C-NMR spectra of compounds CHP5.

**ORIGINAL PHOTOS OF WESTERN BLOT GEL**


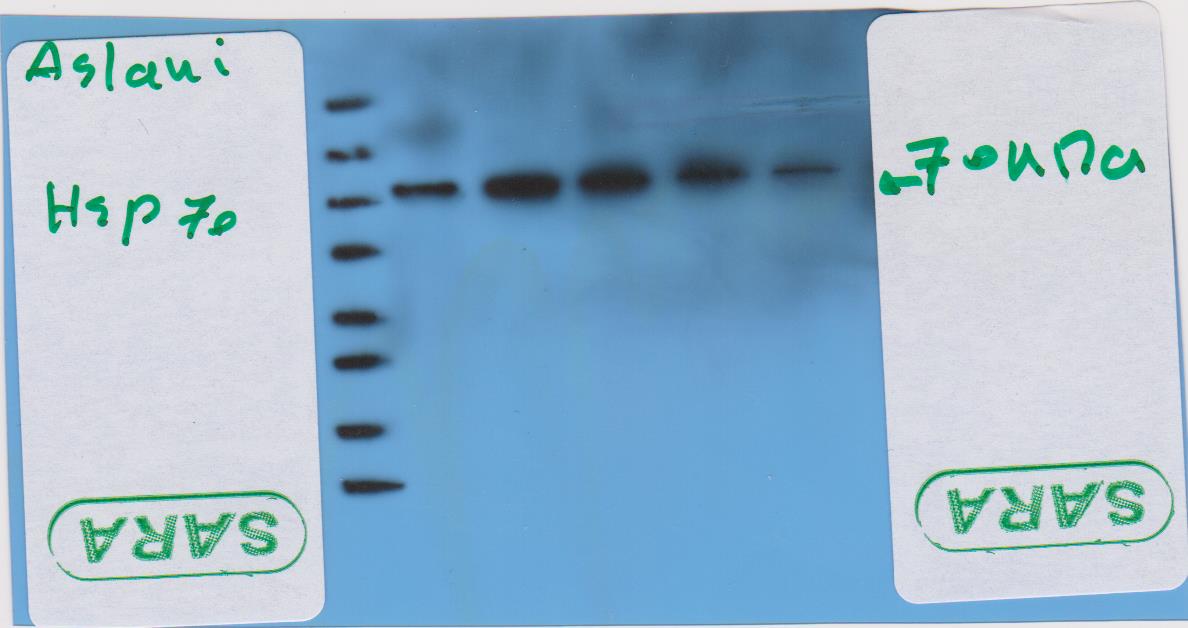


**Fig. S21** Original photo of western blot gel (HSP70). Representative Western blot analysis of HSP70 in the PC12 cell homogenate treated with CHP4 and in the presence of Ab1-42.


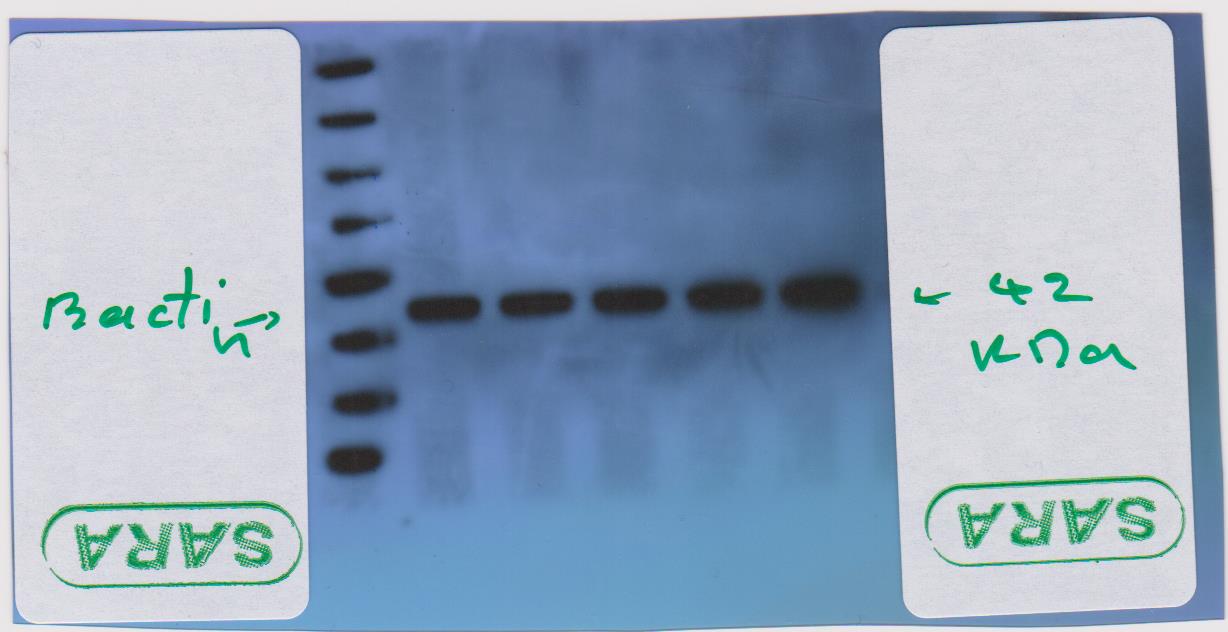


**Fig. S22** Original photo of western blot gel (βactin). Representative Western blot analysis of βactin (control) in the PC12 cell homogenate treated with CHP4 and in the presence of Ab1-42.


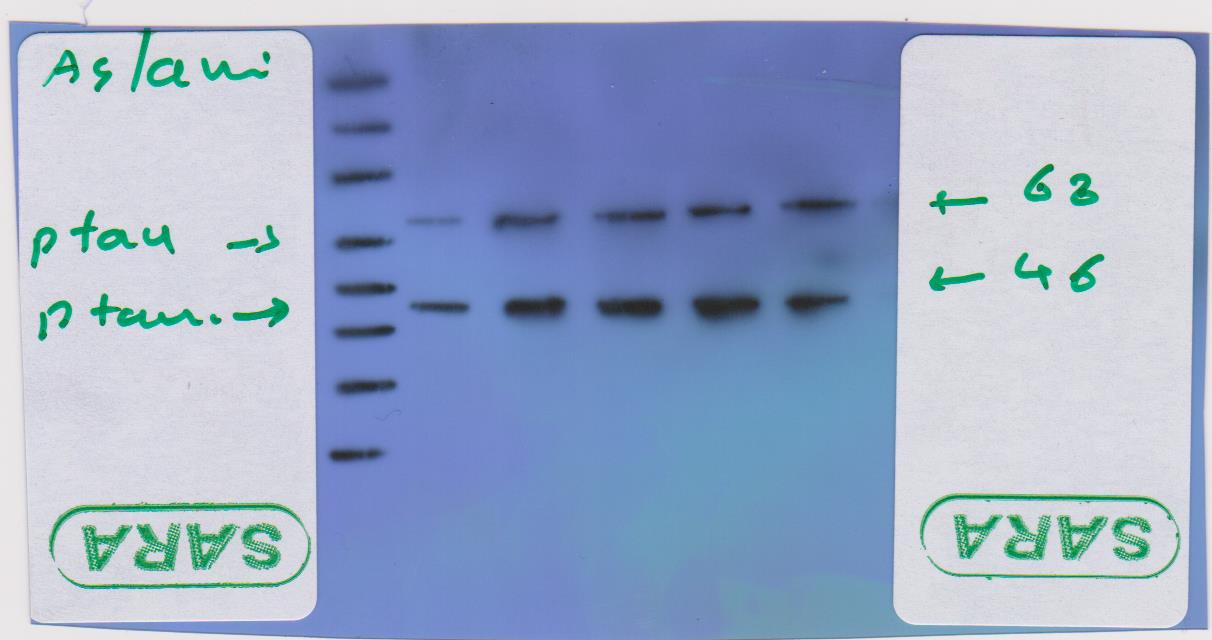


**Fig. S23** Original photo of western blot gel (Tau-p). Representative Western blot analysis of Tau-p (68 and 46 kDa) in the PC12 cell homogenate treated with CHP4 and in the presence of Ab1-42.

**Optimized geometric properties of CHP1 to CHP5**

**Table S1** Optimized geometric properties of CHP1

| **CHP1. Bond length (Å) (DFT)** |  |  |  |
| --- | --- | --- | --- |
| C1-N6 | 1.317 | C17-O21 | 1.346 |
| C1-Cl9 | 1.744 | C1-C2 | 1.426 |
| C2-N3 | 1.339 | C4-C5 | 1.390 |
| C2-N10 | 1.384 | C13-C15 | 1.459 |
| N3-C4 | 1.336 | C15-C16 | 1.484 |
| C5-N6 | 1.341 | C15-C17 | 1.363 |
| N10-H11 | 1.021 | C16-C18 | 1.486 |
| N10-N12 | 1.351 | C18-C22 | 1.400 |
| N12-C13 | 1.287 | C22-C24 | 1.398 |
| C16-O19 | 1.226 | C23-C26 | 1.388 |
| **Bond angle (^◦^) (DFT)** |  |  |  |
| C2-C1-N6 | 120.983 |  |  |
| C2-C1-Cl9 | 122.639 |  |  |
| N6-C1-Cl9 | 116.700 |  |  |
| C1-C2-N10 | 127.289 |  |  |
| N3-C2-N10 | 113.045 |  |  |
| C2-N10-N12 | 124.768 |  |  |
| N10-N12-C13 | 116.038 |  |  |
| N12-C13-C15 | 124.445 |  |  |
| C15-C16-O19 | 124.179 |  |  |
| C17-O21-C22 | 118.920 |  |  |

**Table S2** Optimized geometric properties of CHP2

| **CHP2. Bond length (Å) (DFT)** |  |  |  |
| --- | --- | --- | --- |
| C1-N6 | 1.315 | O25-C26 | 1.424 |
| C1-Cl9 | 1.754 | C1-C2 | 1.427 |
| C2-N3 | 1.341 | C4-C5 | 1.391 |
| C2-N10 | 1.379 | C13-C15 | 1.460 |
| N3-C4 | 1.335 | C15-C16 | 1.409 |
| C5-N6 | 1.342 | C15-C17 | 1.405 |
| N10-H11 | 1.020 | C16-C18 | 1.389 |
| N10-N12 | 1.357 | C17-C20 | 1.393 |
| N12-C13 | 1.287 | C18-C22 | 1.403 |
| C22-O25 | 1.364 | C20-C22 | 1.405 |
| **Bond angle (^◦^) (DFT)** |  |  |  |
| C2-C1-N6 | 121.333 |  |  |
| C2-C1-Cl9 | 122.918 |  |  |
| N6-C1-Cl9 | 115.705 |  |  |
| C1-C2-N10 | 127.495 |  |  |
| N3-C2-N10 | 113.316 |  |  |
| C2-N10-N12 | 124.609 |  |  |
| N10-N12-C13 | 116.843 |  |  |
| N12-C13-C15 | 122.404 |  |  |
| C18-C22-O25 | 115.833 |  |  |
| C22-O25-C26 | 118.781 |  |  |

**Table S3** Optimized geometric properties of CHP3

| **CHP3. Bond length (Å) (DFT)** |  |  |  |
| --- | --- | --- | --- |
| C1-N6 | 1.315 | O25-H26 | 0.966 |
| C1-Cl9 | 1.754 | C1-C2 | 1.429 |
| C2-N3 | 1.343 | C4-C5 | 1.391 |
| C2-N10 | 1.377 | C13-C15 | 1.461 |
| N3-C4 | 1.333 | C15-C16 | 1.411 |
| C5-N6 | 1.341 | C15-C17 | 1.408 |
| N10-H11 | 1.021 | C16-C18 | 1.398 |
| N10-N12 | 1.350 | C17-C20 | 1.393 |
| N12-C13 | 1.289 | C18-C21 | 1.395 |
| C16-O25 | 1.374 | C19-C21 | 1.400 |
| **Bond angle (^◦^) (DFT)** |  |  |  |
| C2-C1-N6 | 121.218 |  |  |
| C2-C1-Cl9 | 122.423 |  |  |
| N6-C1-Cl9 | 115.359 |  |  |
| C1-C2-N10 | 128.455 |  |  |
| N3-C2-N10 | 112.519 |  |  |
| C2-N10-N12 | 126.332 |  |  |
| N10-N12-C13 | 116.672 |  |  |
| N12-C13-C15 | 121.264 |  |  |
| C15-C16-O25 | 117.494 |  |  |
| C16-O25-H26 | 109.834 |  |  |

**Table S4** Optimized geometric properties of CHP4

| **CHP4. Bond length (Å) (DFT)** |  |  |  |
| --- | --- | --- | --- |
| C1-N6 | 1.316 | O25-H26 | 0.966 |
| C1-Cl9 | 1.751 | C1-C2 | 1.426 |
| C2-N3 | 1.340 | C4-C5 | 1.391 |
| C2-N10 | 1.381 | C13-C15 | 1.464 |
| N3-C4 | 1.335 | C15-C16 | 1.407 |
| C5-N6 | 1.341 | C15-C17 | 1.405 |
| N10-H11 | 1.021 | C16-C18 | 1.395 |
| N10-N12 | 1.352 | C17-C20 | 1.393 |
| N12-C13 | 1.287 | C18-C22 | 1.396 |
| C20-O25 | 1.370 | C20-C22 | 1.403 |
| **Bond angle (^◦^) (DFT)** |  |  |  |
| C2-C1-N6 | 121.201 |  |  |
| C2-C1-Cl9 | 122.992 |  |  |
| N6-C1-Cl9 | 115.771 |  |  |
| C1-C2-N10 | 127.581 |  |  |
| N3-C2-N10 | 113.084 |  |  |
| C2-N10-N12 | 124.894 |  |  |
| N10-N12-C13 | 117.069 |  |  |
| N12-C13-C15 | 122.162 |  |  |
| C17-C20-O25 | 117.150 |  |  |
| C20-O25-H26 | 110.087 |  |  |

**Table S5** Optimized geometric properties of CHP5

| **CHP5. Bond length (Å) (DFT)** |  |  |  |
| --- | --- | --- | --- |
| C1-N6 | 1.315 | C1-C2 | 1.425 |
| C1-Cl9 | 1.752 | C4-C5 | 1.391 |
| C2-N3 | 1.340 | C13-C15 | 1.462 |
| C2-N10 | 1.382 | C15-C16 | 1.405 |
| N3-C4 | 1.335 | C15-C17 | 1.408 |
| C5-N6 | 1.341 | C16-C18 | 1.395 |
| N10-H11 | 1.021 | C17-C20 | 1.391 |
| N10-N12 | 1.352 | C18-C22 | 1.394 |
| N12-C13 | 1.287 | C20-C22 | 1.394 |
| C22-Cl25 | 1.757 |  |  |
| **Bond angle (^◦^) (DFT)** |  |  |  |
| C2-C1-N6 | 121.230 |  |  |
| C2-C1-Cl9 | 122.994 |  |  |
| N6-C1-Cl9 | 115.734 |  |  |
| C1-C2-N10 | 127.515 |  |  |
| N3-C2-N10 | 113.136 |  |  |
| C2-N10-N12 | 124.664 |  |  |
| N10-N12-C13 | 117.231 |  |  |
| N12-C13-C15 | 121.875 |  |  |
| C18-C22-C25 | 119.527 |  |  |

**Vina results**

**Table S6** Vina Docking results of CHP4

Mode | Affinity (kcal/mol)

------------+-------------------------

1 -8.3

2 -8.3

3 -8.1

4 -8.0

5 -7.9

6 -7.6

**Table S7** Vina Docking results of DPZ (donepezil)

Mode | Affinity (kcal/mol

------------+----------------------

1 -12.8

2 -11.3

3 -10.9

4 -10.5

5 -10.4

6 -10.3

**Diagram of capability of the synthesized compounds in passing from BBB**


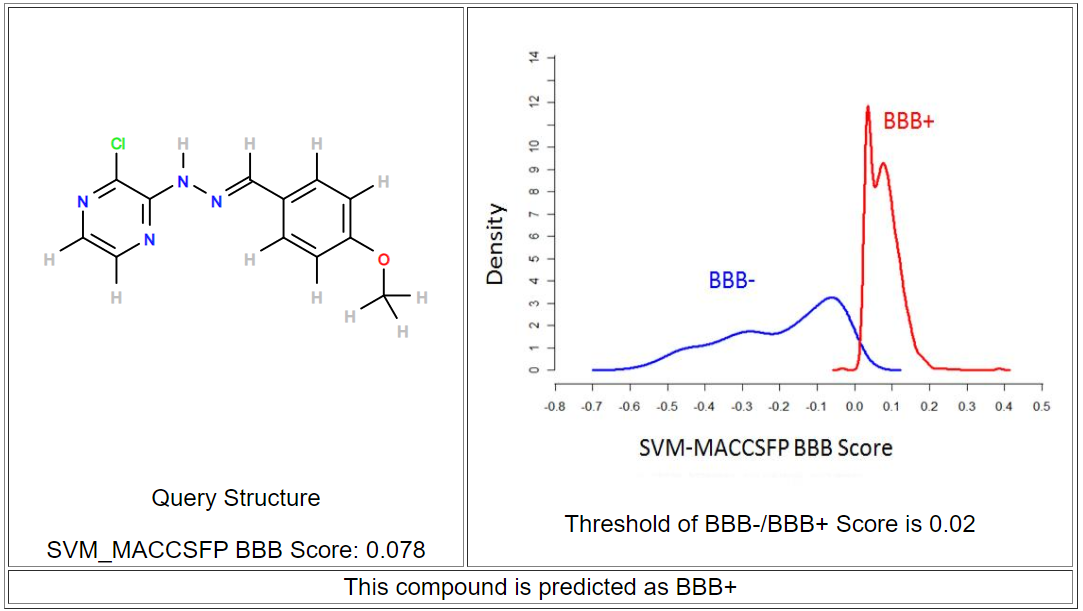


**Fig. S24** Diagram of capability of the CHP1 in passing from BBB


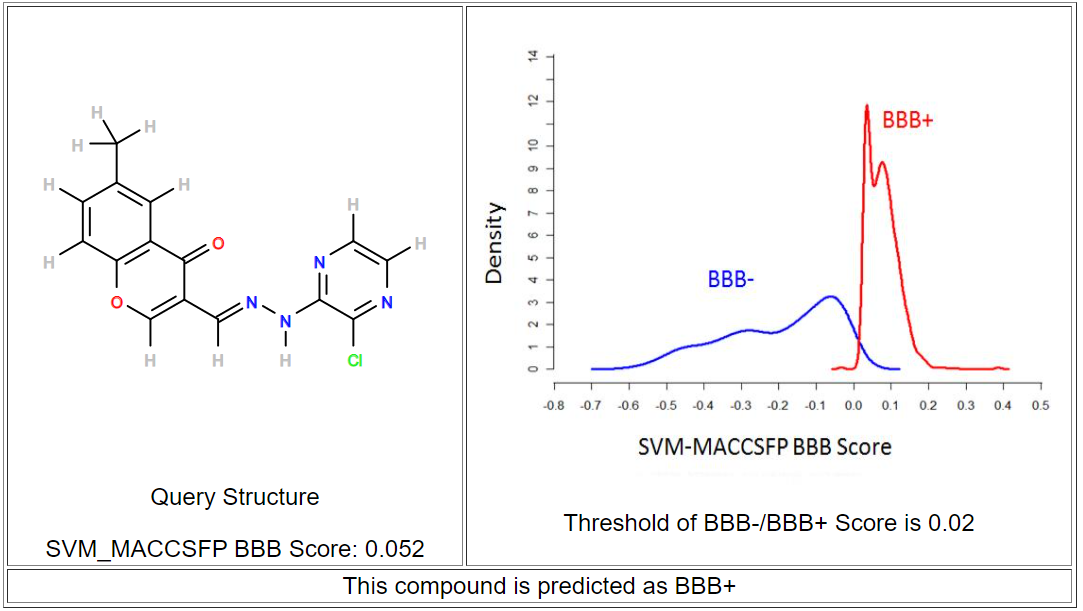


**Fig. S25** Diagram of capability of the CHP2 in passing from BBB


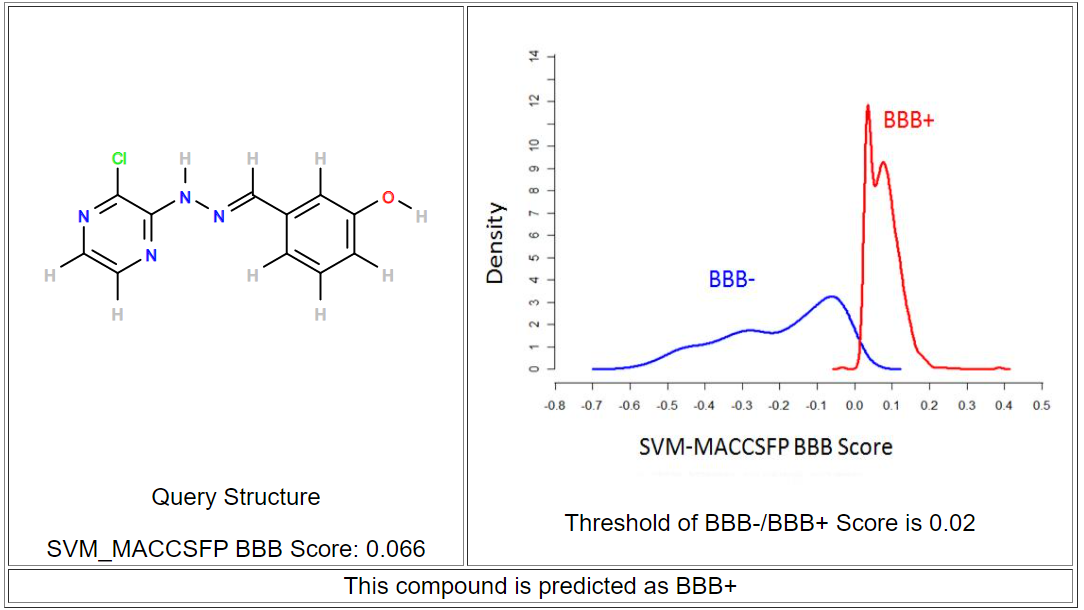


**Fig. S26** Diagram of capability of the CHP3 in passing from BBB


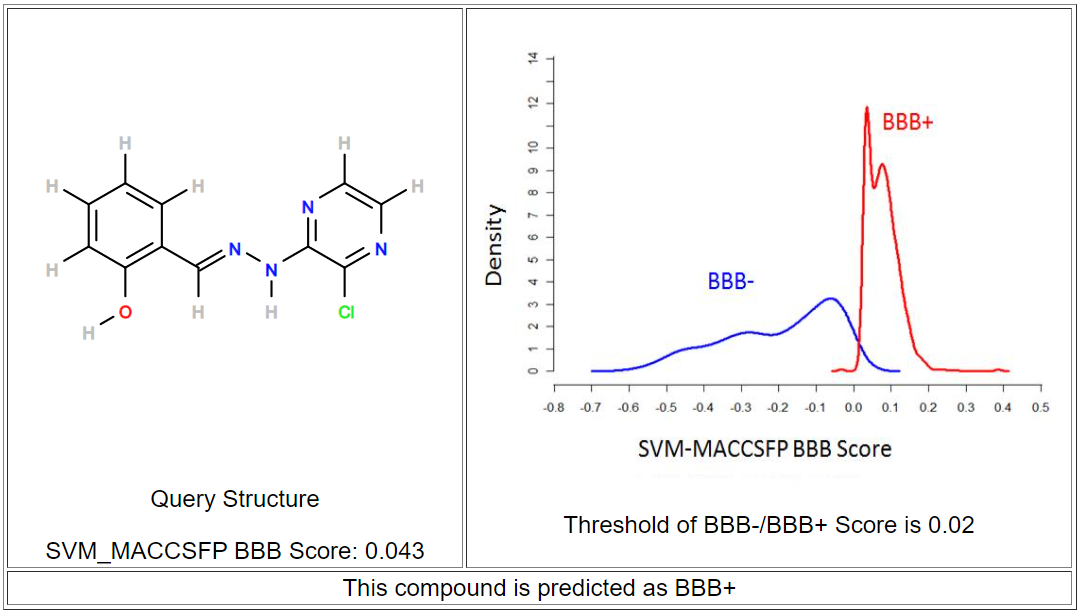


**Fig. S27** Diagram of capability of the CHP4 in passing from BBB


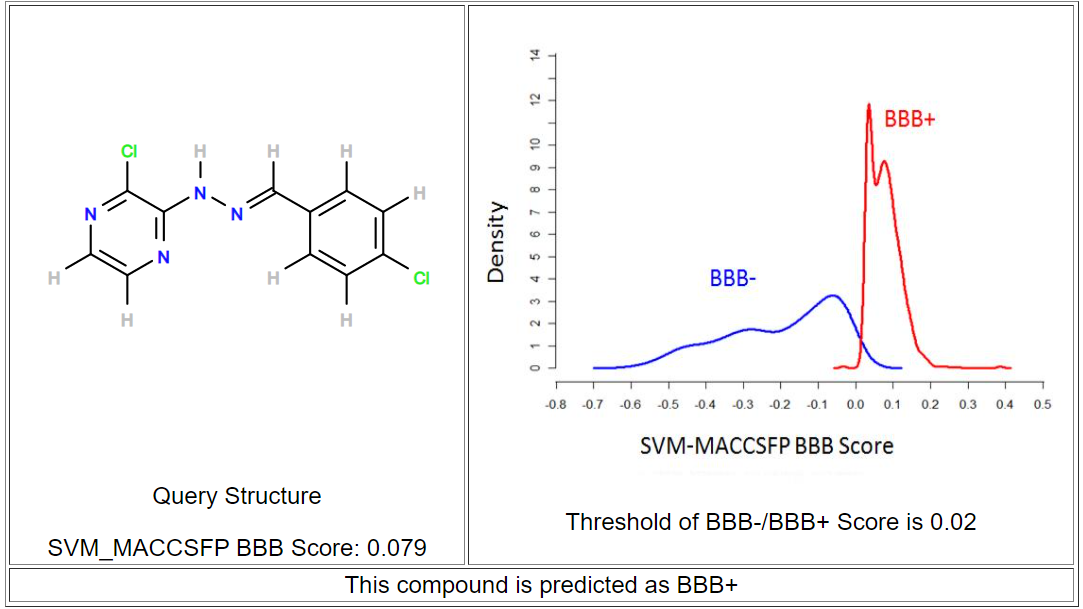


**Fig. S28** Diagram of capability of the CHP5 in passing from BBB

**Protein and ligand intraction**


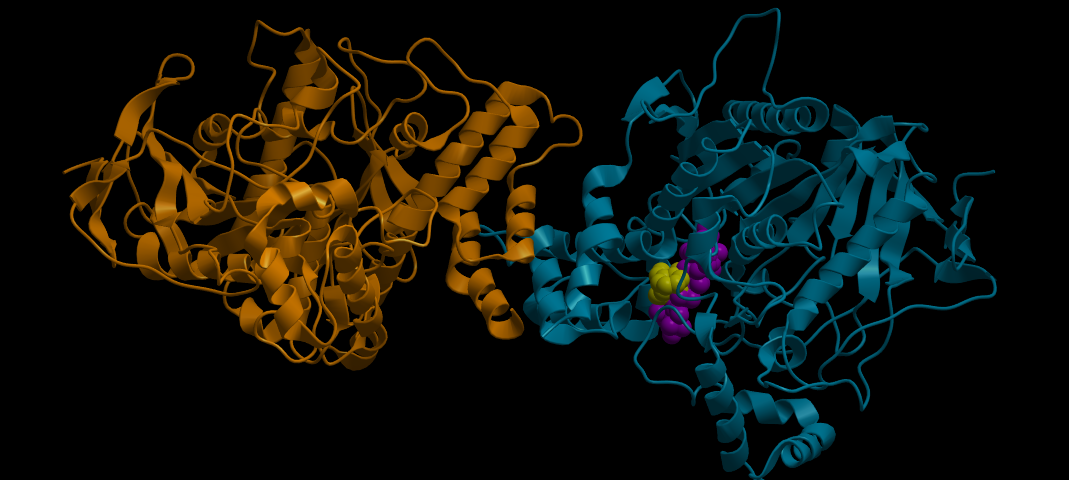


**Fig. S29** Ribbon model of the dimeric structure of AChE, chain an AChE (orange); chain b (blue). Docked pose of CHP4 (yellow) occupy the same Cartesian space as DPZ (violet). The CHP4 overlapped with DPZ in the AChE-binding.


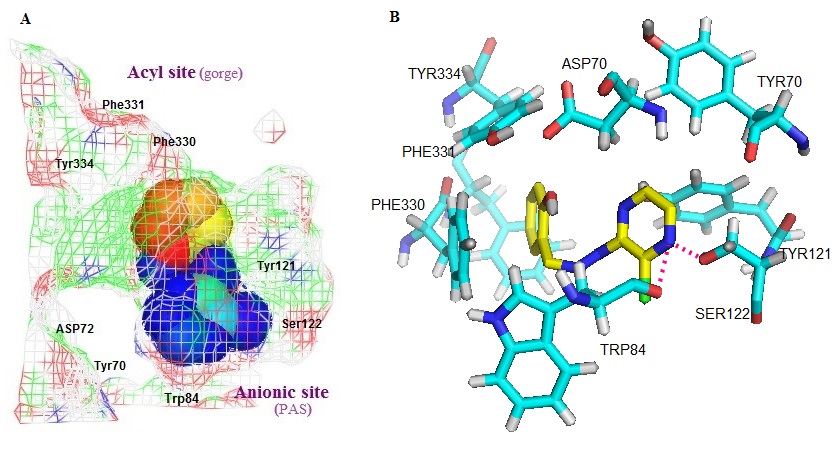


**Fig. S30** Representation a binding pocket of AChE. (A) Interaction of aromatic amino acids and ligand in the binding cleft; (B) Magnified the active site of the AChE (cyan) and CPH4 (yellow) complex, exhibiting segment of CHP4 together with AChE in the active site. Several important residues in the binding gorge are demonstrated using PyMOL.
